# Efficacy of immune-based combinations across treatment lines in advanced hepatocellular carcinoma: a systematic review and network meta-analysis

**DOI:** 10.3389/fphar.2026.1837089

**Published:** 2026-06-10

**Authors:** Wenbin Zhao, Zhichao Wu, Shengxian Qiao, Qingyan Kou, Zhenyuan Liu, Xu Zhang

**Affiliations:** Qingdao Central Hospital, University of Health and Rehabilitation Sciences, Qingdao, China

**Keywords:** hepatocellular carcinoma, immunotherapy, network meta-analysis, systemic treatment, targeted therapy

## Abstract

**Background:**

With the rapid introduction of immune checkpoint inhibitors (ICIs) for advanced hepatocellular carcinoma (HCC), optimal treatment sequencing remains unclear. Lacking direct comparisons, we aimed to evaluate the efficacy and safety of systemic therapies across first- and second-line settings.

**Methods:**

A frequentist network meta-analysis (PROSPERO: CRD420261296427) was performed using phase III RCTs from PubMed, Embase, Cochrane, and Web of Science (up to January 2026) evaluating systemic HCC therapies. The primary endpoint was overall survival (OS); secondary endpoints included progression-free survival (PFS), objective response rate (ORR), and Grade ≥3 treatment-related adverse events (TRAEs). Subgroup analysis compared treatment-naïve versus refractory populations.

**Results:**

Twelve RCTs comprising 8,138 patients were analyzed. For OS, ICI-anti-angiogenic combinations ranked highest, notably sintilimab plus IBI305 (HR = 0.57 vs. sorafenib; SUCRA = 0.94) and camrelizumab plus rivoceranib (HR = 0.62 vs. sorafenib; SUCRA = 0.89). Combinations consistently outperformed monotherapies in PFS and ORR. Crucially, subgroup analysis revealed a statistically significant difference in the magnitude of survival benefit between first-line (HR = 0.74, 95% CI: 0.65–0.83) and second-line settings (HR = 1.09, 95%CI: 0.90–1.30) when compared to sorafenib (P = 0.0006). Regarding safety, ICI monotherapy/dual-blockade (e.g., pembrolizumab, nivolumab + ipilimumab) demonstrated better tolerability, whereas TKI-based combinations significantly increased Grade ≥3 TRAE rates.

**Conclusion:**

ICI-based combinations offer the most robust survival benefits in HCC via pharmacodynamic synergy, albeit with higher cumulative toxicity. The differing magnitude of survival benefit between first- and second-line settings when compared to sorafenib highlights their distinct clinical contexts. These findings support a tailored continuum of care, guiding optimal sequencing based on pharmacological efficacy and safety profiles.

**Systematic Review Registration:**

https://www.crd.york.ac.uk/PROSPERO/view/CRD420261296427, identifier CRD420261296427.

## Introduction

Primary liver cancer remains a significant global health burden, ranking as the sixth most commonly diagnosed cancer and the third leading cause of cancer-related mortality worldwide (Sung et al., 2021). Hepatocellular carcinoma (HCC) accounts for approximately 75%–85% of primary liver cancer cases (Rumgay et al., 2022). Due to the insidious onset of the disease, the majority of patients are diagnosed at an advanced stage, where curative treatments such as surgical resection, ablation, or liver transplantation are no longer feasible. For these patients, systemic therapy represents the mainstay of management to prolong survival and maintain quality of life (Llovet et al., 2021).

For over a decade, the multikinase inhibitor (MKI) sorafenib was the sole standard-of-care for advanced HCC, based on the pivotal SHARP trial (Llovet et al., 2008). This landscape has evolved rapidly in recent years. Lenvatinib demonstrated non-inferiority to sorafenib in the REFLECT trial, becoming another first-line option (Kudo et al., 2018). Subsequently, the advent of immune checkpoint inhibitors (ICIs) has revolutionized the treatment paradigm. The combination of atezolizumab plus bevacizumab (IMbrave150) established a new benchmark for overall survival (OS), superior to sorafenib (Finn et al., 2020a). More recently, the HIMALAYA trial introduced the dual immunotherapy regimen of tremelimumab plus durvalumab (STRIDE regimen) as a standard first-line option (Abou-Alfa et al., 2022).

Despite these advancements in the first-line setting, disease progression is inevitable for most patients. The prognosis for patients with refractory or progressed HCC remains poor, highlighting an urgent need for effective second-line and later-line therapies (Pinter et al., 2021a). Several TKIs, including regorafenib (Bruix et al., 2017) and cabozantinib (Abou-Alfa et al., 2018), and the anti-VEGFR2 antibody ramucirumab (for patients with alpha-fetoprotein [AFP] ≥400 ng/mL) (Zhu et al., 2019), have demonstrated survival benefits compared with placebo in the second-line setting. Additionally, PD-1 inhibitors such as pembrolizumab have shown promising activity. Although the global phase III KEYNOTE-240 trial narrowly missed its primary endpoints (Finn et al., 2020b), the subsequent KEYNOTE-394 trial confirmed the significant survival benefit of pembrolizumab in Asian patients (Qin et al., 2022).

Furthermore, the therapeutic landscape is becoming increasingly complex with the emergence of novel combination strategies tested in both first-line and refractory settings. Recent phase III trials, such as CARES-310 (camrelizumab plus rivoceranib) (Qin et al., 2023), ORIENT-32 (sintilimab plus IBI305) (Ren et al., 2021), and CheckMate 9DW (nivolumab plus ipilimumab) (Yau et al., 2025), have reported substantial efficacy data. Conversely, some combinations, such as lenvatinib plus pembrolizumab in the LEAP-002 trial, failed to meet statistical significance for OS improvement (Llovet et al., 2023). Indeed, while ICI-based combinations hold unprecedented curative potential for HCC, optimizing these regimens and overcoming the highly immunosuppressive liver microenvironment remain ongoing challenges (Tong et al., 2025). With the proliferation of therapeutic agents, including monotherapies and various combinations, clinicians face a dilemma in selecting the optimal treatment sequence (Vogel et al., 2021).

Currently, there are no head-to-head randomized controlled trials (RCTs) directly comparing these diverse active systemic therapies, particularly comparing the newer immune-based combinations against the established standard of care, sorafenib, in a unified framework. Consequently, the relative efficacy and safety of these regimens remain uncertain (Sonbol et al., 2020; Fulgenzi et al., 2023). To address this evidence gap, we conducted a systematic review and network meta-analysis (NMA) of randomized phase III trials. This study aims to compare the efficacy and safety of available systemic therapies for advanced HCC, incorporating the most recent evidence to guide clinical decision-making.

## Methods

### Study design and registration

This systematic review and network meta-analysis (NMA) was conducted in accordance with the Preferred Reporting Items for Systematic Reviews and Meta-Analyses (PRISMA) extension statement for Network Meta-Analyses (Hutton et al., 2015) and was prospectively registered in PROSPERO (CRD420261296427). The study protocol was designed to evaluate systemic therapies for advanced hepatocellular carcinoma (HCC), focusing on both refractory (second-line) and treatment-naïve (first-line) populations to provide a comprehensive landscape of therapeutic efficacy.

### Data sources and search strategy

We performed a comprehensive search of PubMed/MEDLINE, Embase, the Cochrane Central Register of Controlled Trials (CENTRAL), and Web of Science from inception to January 2026. We also manually screened conference proceedings from major oncology meetings (ASCO, ESMO, AACR) to identify relevant unpublished data. The search strategy combined Medical Subject Headings (MeSH) and free-text terms related to “hepatocellular carcinoma,” “systemic therapy,” “immunotherapy,” “targeted therapy,” and “randomized controlled trial.” No language restrictions were applied.

## Selection criteria

### Studies were included if they met the following criteria

Population: Adult patients (≥18 years) with histologically or clinically confirmed advanced HCC who were either treatment-naïve or had progressed after prior systemic therapy.

Intervention: Systemic therapies, including tyrosine kinase inhibitors (TKIs), immune checkpoint inhibitors (ICIs), anti-angiogenic agents, or their combinations.

Comparator: Placebo, best supportive care (BSC), or an active comparator (e.g., sorafenib, lenvatinib).

Outcomes: Reported at least one of the following: Overall Survival (OS), Progression-Free Survival (PFS), Objective Response Rate (ORR), or safety data (Grade ≥3 Treatment-Related Adverse Events [TRAEs]).

Study Design: Randomized controlled trials (RCTs). Single-arm studies, observational studies, and editorials were excluded.

### Data extraction and quality assessment

Two independent reviewers extracted data using a standardized predefined form. Extracted data included study characteristics (author, year, phase, region), patient demographics (age, sex, etiology, BCLC stage, AFP levels), treatment details, and outcome measures. For survival outcomes (OS and PFS), hazard ratios (HRs) and 95% confidence intervals (CIs) were extracted. For dichotomous outcomes (ORR and safety), the number of events and total number of patients were recorded.

The methodological quality of the included RCTs was assessed using the Cochrane Risk of Bias tool version 2 (RoB 2) (Sterne et al., 2019). Domains assessed included randomization process, deviations from intended interventions, missing outcome data, measurement of the outcome, and selection of the reported result. Studies were classified as having “low risk,” “some concerns,” or “high risk” of bias.

### Statistical analysis

We performed a network meta-analysis within a frequentist framework using the netmeta package (version 2.8-0) in R software (version 4.3.0) (Balduzzi et al., 2023). Sorafenib was set as the common reference treatment for all network comparisons, as it represents the established standard of care for advanced HCC.

Effect Measures: For time-to-event outcomes (OS and PFS), the treatment effects were expressed as Hazard Ratios (HRs) with 95% CIs. For binary outcomes (ORR and Grade ≥3 TRAEs), Odds Ratios (ORs) with 95% CIs were calculated.

Network Geometry: Network plots were generated to visualize the comparative evidence, where node size corresponds to the number of patients and edge thickness represents the number of trials.

Data Synthesis: A random-effects model was employed for all analyses to account for potential between-study heterogeneity. Heterogeneity was quantified using the I^2^ statistic and the Τ^2^ (tau-squared) variance estimator.

Ranking of Treatments: To estimate the hierarchy of competing interventions, we calculated the Surface Under the Cumulative Ranking Curve (SUCRA) and P-scores. SUCRA values range from 0 to 1, where a higher value indicates a higher likelihood that a therapy is the most effective (or safest) (Salanti et al., 2011).

Subgroup and Sensitivity Analysis: Given the inclusion of both first-line and second-line studies, a pre-specified subgroup analysis was performed to evaluate the impact of the line of therapy on treatment efficacy. Interaction tests were used to assess statistical differences between subgroups.

Consistency and Publication Bias: Inconsistency between direct and indirect evidence was assessed using the node-splitting method where closed loops existed. Publication bias was evaluated visually using comparison-adjusted funnel plots (Chaimani and Salanti, 2015).

All statistical tests were two-sided, and a P-value of <0.05 was considered statistically significant.

## Results

### Study selection and characteristics

**FIGURE 1:**
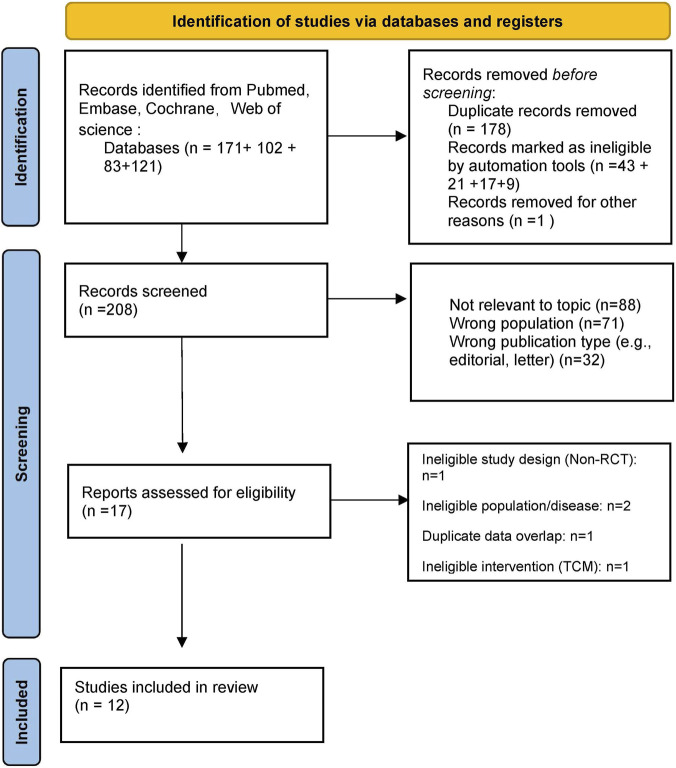
PRISMA flow diagram of study selection.

The comprehensive literature search identified a total of 477 records from electronic databases. After records were removed before screening, 208 records were screened. Seventeen full-text reports were assessed for eligibility, and 12 randomized controlled trials were finally included in the network meta-analysis (Figure 1).

These 12 RCTs comprised 8,138 patients with advanced hepatocellular carcinoma (HCC). The network included four studies conducted in the second-line setting (RESORCE, CELESTIAL, REACH-2, KEYNOTE-394) and eight studies in the first-line setting (IMbrave150, REFLECT, HIMALAYA, ORIENT-32, CARES-310, CheckMate 9DW, LEAP-002, and SHARP). The SHARP trial served as a critical bridge connecting the sorafenib-based first-line network with the placebo-controlled second-line network.

The baseline characteristics of the included studies are summarized in Table 1. The median age of participants ranged from 53 to 66 years. The prevalence of Hepatitis B Virus (HBV) infection varied significantly by region, ranging from 19% in global studies (e.g., SHARP) to 94% in Asian-predominant studies (e.g., ORIENT-32). Most patients had Barcelona Clinic Liver Cancer (BCLC) stage C disease and preserved liver function (Child-Pugh A).

**TABLE 1:** Baseline characteristics of included randomized controlled trials.

Study	Year	Phase	Setting	Treatment_Arms	Total_N	Median_Age	Male_Percent	HBV_Etiology	HCV_Etiology	BCLC_C_Stage	AFP_ge_400
RESORCE	2017	III	2nd line	Regorafenib vs. placebo	573	63	88%	38%	21%	87%	43%
CELESTIAL	2018	III	2nd line	Cabozantinib vs. placebo	707	64	82%	38%	24%	81%	41%
REACH-2	2019	III	2nd line	Ramucirumab vs. placebo	292	64	78%	36%	26%	82%	100%
KEYNOTE-394	2022	III	2nd line	Pembrolizumab vs. placebo	453	54	85%	79%	2%	93%	34%
SHARP	2008	III	1st line	Sorafenib vs. placebo	602	65	87%	19%	28%	82%	30%
IMbrave150	2020	III	1st line	Atezo + Bev vs. sorafenib	501	64	82%	49%	21%	82%	38%
REFLECT	2018	III	1st line	Lenvatinib vs. sorafenib	954	62	84%	53%	19%	79%	46%
HIMALAYA	2022	III	1st line	Durva + Treme vs. sorafenib	1,171	65	83%	31%	27%	86%	40%
ORIENT-32	2021	II/III	1st line	Sinti + IBI305 vs. sorafenib	571	53	88%	94%	2%	85%	43%
CARES-310	2023	III	1st line	Camre + Rivo vs. sorafenib	543	58	84%	76%	8%	86%	35%
CheckMate 9DW	2025	III	1st line	Nivo + Ipi vs. len/Sora	668	66	81%	34%	28%	73%	32%
LEAP-002	2023	III	1st line	Len + Pembro vs. Len + Pbo	794	66	81%	49%	24%	78%	31%

Abbreviations: AFP, alpha-fetoprotein; BCLC, Barcelona Clinic Liver Cancer; ECOG PS, Eastern Cooperative Oncology Group performance status; HBV, hepatitis B virus; HCV, hepatitis C virus; N, number of patients. Data are presented as median (range) or number (%) unless otherwise indicated.

### Risk of bias assessment

**FIGURE 2:**
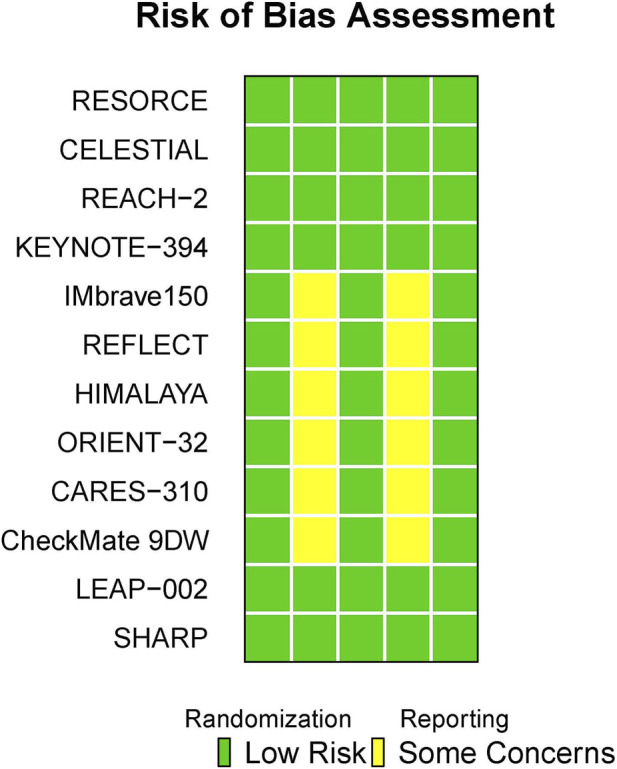
Risk of bias assessment. Traffic light plot presenting the risk of bias for included randomized controlled trials. Studies are rated as “Low Risk” (green) or “Some Concerns” (yellow) for the domains displayed in the figure.

The risk of bias assessment using the Cochrane RoB 2.0 tool is presented in Figure 2. The majority of second-line trials (e.g., RESORCE, CELESTIAL) were double-blind and rated as “Low Risk” across all domains. Several first-line trials (e.g., CheckMate 9DW, IMbrave150) were open-label, leading to a rating of “Some Concerns” in the domains of deviations from intended interventions and outcome measurement, particularly for subjective outcomes. Overall, the quality of the included evidence was considered robust.

### Network geometry

**FIGURE 3:**
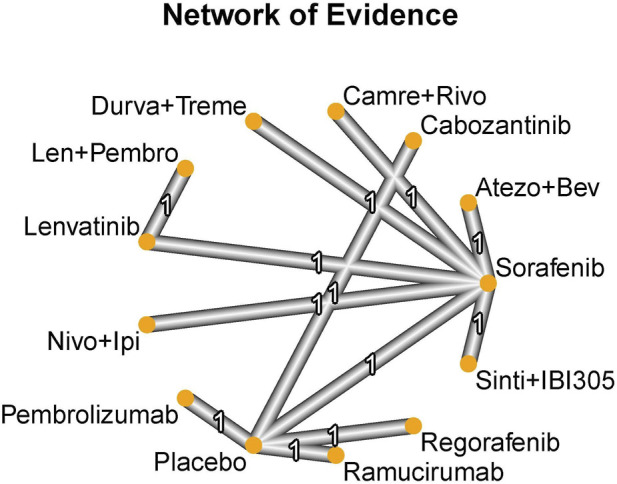
Network Geometry of Comparisons for Overall Survival. The network plot represents the evidence base for overall survival (OS). The nodes (circles) represent the competing treatments, and the edges (lines) represent direct head-to-head comparisons between interventions. The size of the nodes is proportional to the number of patients randomised to each treatment, and the thickness of the lines is proportional to the number of trials comparing the connected treatments.

The network geometry for the primary endpoint, Overall Survival (OS), is illustrated in Figure 3. The network formed a star-shaped pattern with multiple active interventions compared against common comparators (Placebo or Sorafenib). The nodes for Sorafenib and Placebo were the most connected, ensuring the connectivity of the entire network.

### Overall survival (primary endpoint)

**FIGURE 4:**
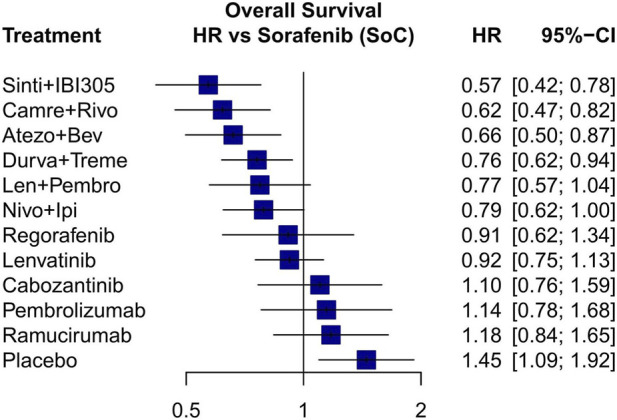
Forest Plot of Overall Survival (vs. Sorafenib). Forest plot summarizing the Hazard Ratios (HRs) and 95% Confidence Intervals (CIs) for overall survival of all active treatments compared with Sorafenib. HR < 1 indicates a survival benefit favoring the active treatment. The treatments are sorted by effect size.

The forest plot (Figure 4) and league table (Table 2) show that immune-checkpoint inhibitor (ICI) combinations, such as Sintilimab plus IBI305 (HR 0.57, 95% CI 0.42–0.78) and Camrelizumab plus Rivoceranib (HR 0.62, 95% CI 0.47–0.82), exhibited the most profound risk reduction.

**TABLE 2:** League table of aggregate relative effect sizes for Overall Survival and Progression-Free Survival.

Treatment	Atezo + Bev	Cabozantinib	Camre + Rivo	Durva + Treme	Len + Pembro	Lenvatinib	Nivo + Ipi	Pembrolizumab	Placebo	Ramucirumab	Regorafenib	Sinti + IBI305	Sorafenibl
Atezo + Bev	Atezo + Bev	​	​	​	​	​	​	​	​	​	​	​	0.66 (0.52–0.84)
Cabozantinib	0.60 (0.40–0.89)	Cabozantinib	​	​	​	​	​	​	0.76 (0.63–0.92)	​	​	​	​
Camre + Rivo	1.06 (0.75–1.51)	1.78 (1.20–2.63)	Camre + Rivo	​	​	​	​	​	​	​	​	​	0.62 (0.49–0.79)
Durva + Treme	0.87 (0.65–1.16)	1.45 (1.03–2.04)	0.82 (0.61–1.09)	Durva + Treme	​	​	​	​	​	​	​	​	0.76 (0.65–0.89)
Len + Pembro	0.85 (0.61–1.19)	1.43 (0.97–2.08)	0.80 (0.57–1.12)	0.98 (0.75–1.29)	Len + Pembro	0.84 (0.71–1.00)	​	​	​	​	​	​	​
Lenvatinib	0.72 (0.54–0.96)	1.20 (0.85–1.68)	0.67 (0.51–0.90)	0.83 (0.67–1.02)	0.84 (0.71–1.00)	Lenvatinib	​	​	​	​	​	​	0.92 (0.79–1.07)
Nivo + Ipi	0.84 (0.61–1.14)	1.39 (0.97–2.00)	0.78 (0.57–1.07)	0.96 (0.75–1.24)	0.98 (0.73–1.32)	1.16 (0.91–1.49)	Nivo + Ipi	​	​	​	​	​	0.79 (0.65–0.96)
Pembrolizumab	0.58 (0.38–0.87)	0.96 (0.72–1.29)	0.54 (0.36–0.82)	0.66 (0.46–0.96)	0.67 (0.45–1.01)	0.80 (0.56–1.15)	0.69 (0.47–1.01)	Pembrolizumab	0.79 (0.63–0.99)	​	​	​	​
Placebo	0.46 (0.32–0.64)	0.76 (0.63–0.92)	0.43 (0.30–0.60)	0.52 (0.39–0.70)	0.53 (0.38–0.74)	0.63 (0.48–0.84)	0.55 (0.40–0.74)	0.79 (0.63–0.99)	Placebo	1.41 (1.05–1.89)	1.59 (1.26–2.00)	​	1.45 (1.14–1.84)
Ramucirumab	0.64 (0.41–1.01)	1.07 (0.76–1.52)	0.60 (0.38–0.95)	0.74 (0.49–1.11)	0.75 (0.48–1.17)	0.89 (0.60–1.34)	0.77 (0.50–1.17)	1.11 (0.77–1.61)	1.41 (1.05–1.89)	Ramucirumab	​	​	​
Regorafenib	0.72 (0.48–1.09)	1.21 (0.90–1.62)	0.68 (0.45–1.03)	0.83 (0.58–1.20)	0.85 (0.57–1.26)	1.01 (0.70–1.45)	0.87 (0.59–1.27)	1.25 (0.91–1.73)	1.59 (1.26–2.00)	1.13 (0.78–1.63)	Regorafenib	​	​
Sinti + IBI305	1.16 (0.80–1.68)	1.93 (1.28–2.92)	1.09 (0.75–1.58)	1.33 (0.97–1.84)	1.36 (0.95–1.94)	1.61 (1.18–2.21)	1.39 (0.99–1.95)	2.01 (1.30–3.09)	2.54 (1.76–3.67)	1.81 (1.13–2.89)	1.60 (1.04–2.47)	Sinti + IBI305	0.57 (0.43–0.75)
Sorafenib	0.66 (0.52–0.84)	1.10 (0.81–1.50)	0.62 (0.49–0.79)	0.76 (0.65–0.89)	0.77 (0.62–0.97)	0.92 (0.79–1.07)	0.79 (0.65–0.96)	1.14 (0.82–1.59)	1.45 (1.14–1.84)	1.03 (0.70–1.50)	0.91 (0.66–1.27)	0.57 (0.43–0.75)	Sorafenib

**FIGURE 5:**
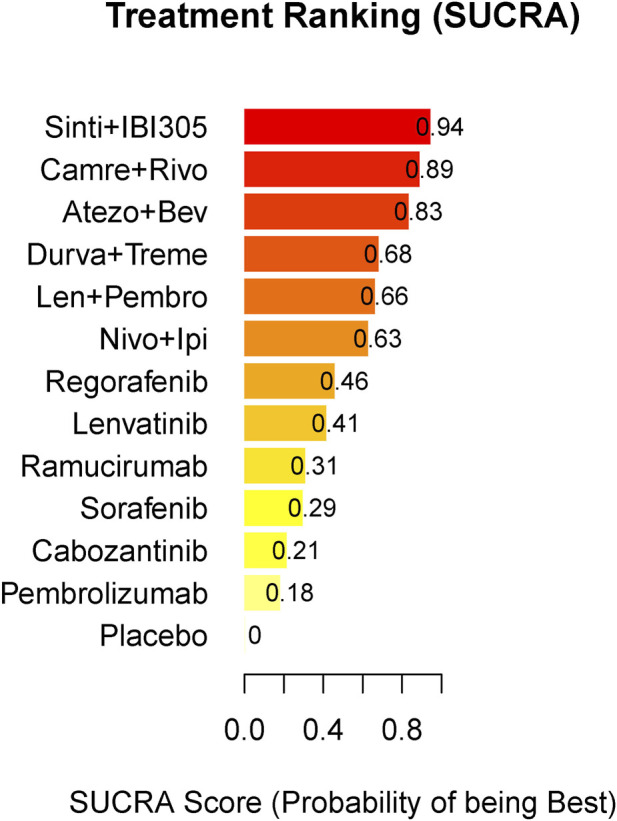
SUCRA Ranking Probabilities for Overall Survival Ranking of treatments for overall survival based on the Surface Under the Cumulative Ranking Curve (SUCRA). Higher SUCRA scores (closer to 1.0) indicate a higher probability of the treatment being the most effective in prolonging survival.

According to the Surface Under the Cumulative Ranking Curve (SUCRA) (Figure 5; Table 3), Sintilimab plus IBI305 ranked highest (SUCRA = 0.94), followed by Camrelizumab plus Rivoceranib (SUCRA = 0.89) and Atezolizumab plus Bevacizumab (SUCRA = 0.83). Among the monotherapies, Pembrolizumab (SUCRA = 0.18) and Sorafenib (SUCRA = 0.29) ranked lower than combination therapies.

**TABLE 3:** SUCRA ranking probabilities of treatments for efficacy and safety outcomes.

Treatment	SUCRA_OS
Atezo + Bev	0.833
Cabozantinib	0.214
Camre + Rivo	0.889
Durva + Treme	0.679
Len + Pembro	0.661
Lenvatinib	0.415
Nivo + Ipi	0.627
Pembrolizumab	0.179
Placebo	0.003
Ramucirumab	0.307
Regorafenib	0.457
Sinti + IBI305	0.942
Sorafenib	0.294

### Subgroup analysis: first-line vs. second-line

**FIGURE 6:**
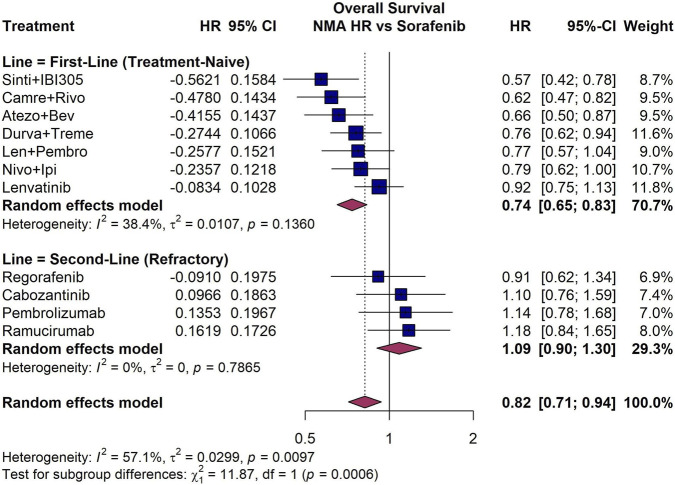
Subgroup Analysis: First-line vs. Second-line Treatments Subgroup analysis comparing the magnitude of Overall Survival benefit (Hazard Ratio) between First-line (Treatment-Naïve) and Second-line (Refractory) studies. The diamond represents the pooled effect estimate for each subgroup. The test for subgroup differences (P < 0.01) indicates statistically significant difference in relative efficacy against Sorafenib between the two lines of therapy.

To address potential heterogeneity arising from mixing treatment lines, a subgroup analysis was performed (Figure 6). The pooled Hazard Ratio for active treatments versus sorafenib was 0.74 (95% CI 0.65–0.83) in first-line studies (I2 = 38.4%) and 1.09 (95% CI 0.90–1.30) in second-line studies (I^2^ = 0%). The test for subgroup differences yielded a P-value of 0.0006, indicating a statistically significant difference in the magnitude of survival benefit provided by active agents across treatment lines when compared to sorafenib.

### Progression-free survival (PFS)

The network for PFS is shown in Supplementary Figure S1A. The relative efficacy of active treatments for PFS compared with sorafenib is summarized in the forest plot (Supplementary Figure S1B). Consistent with OS results, the SUCRA ranking for PFS (Supplementary Figure S2; Table 3) favored combination therapies, with Camrelizumab plus Rivoceranib (SUCRA = 0.93) and Sintilimab plus IBI305 (SUCRA = 0.87) ranking highest. Lenvatinib plus Pembrolizumab (SUCRA = 0.85) also demonstrated high efficacy in delaying disease progression.

### Objective response rate (ORR)

The network geometry for ORR is presented in Supplementary Figure S3A. The relative odds of achieving an objective response compared with sorafenib are summarized in Supplementary Figure S3B. The SUCRA ranking (Supplementary Figure S4; Table 3) indicated that Camrelizumab plus Rivoceranib (SUCRA = 0.86) and Sintilimab plus IBI305 (SUCRA = 0.80) were the most effective in inducing tumor shrinkage. Notably, dual immunotherapy (Nivolumab plus Ipilimumab) and Lenvatinib-based regimens also ranked highly.

### Safety (grade ≥ 3 TRAEs)

Safety was assessed based on the incidence of Grade ≥3 treatment-related adverse events (TRAEs) (Supplementary Figure S5). In the SUCRA ranking for safety (where higher scores indicate a better safety profile), Placebo ranked highest (SUCRA = 0.98), as expected (Supplementary Figure S6; Table 3). Among active treatments, immunotherapy monotherapy (Pembrolizumab, SUCRA = 0.82) and dual immunotherapy (Nivolumab plus Ipilimumab, SUCRA = 0.56) showed better tolerability compared to TKI-based combinations. Camrelizumab plus Rivoceranib (SUCRA = 0.01) and Sintilimab plus IBI305 (SUCRA = 0.12) had the lowest probability of being the safest, indicating a higher risk of severe adverse events associated with these potent combination regimens.

### Publication bias

The comparison-adjusted funnel plot for overall survival (Supplementary Figure S7) showed a generally symmetrical distribution of studies around the null line, suggesting a low risk of small-study effects or publication bias within the network.

## Discussion

This network meta-analysis provides a comprehensive synthesis of randomized controlled trials evaluating systemic therapies for advanced hepatocellular carcinoma (HCC). By integrating data from 12 pivotal trials involving over 8,000 patients, we established a hierarchy of efficacy and safety for current standard-of-care regimens. Our primary findings indicate that immune checkpoint inhibitor (ICI)-based combinations, particularly the dual immunotherapy regimen (nivolumab plus ipilimumab) and ICI plus anti-angiogenic TKI combinations (e.g., camrelizumab plus rivoceranib, sintilimab plus IBI305), offer the most robust overall survival (OS) and progression-free survival (PFS) benefits compared with sorafenib, the established first-line standard of care. Importantly, our subgroup analysis anchored to sorafenib revealed a statistically significant difference between first-line and second-line settings (P = 0.0006). This underscores that while first-line combinations demonstrate profound superiority over sorafenib, the relative efficacy of second-line therapies appears different when directly compared to an active first-line baseline, reflecting their distinct clinical contexts in refractory populations.

The superiority of ICI-based combinations observed in our study is deeply rooted in the pharmacodynamic synergy between anti-angiogenic agents and immune checkpoint blockade, aligning with the current understanding of the tumor microenvironment (TME) in HCC (Tong et al., 2025). Hepatocellular carcinoma is characterized by a highly immunosuppressive TME and aberrant vascularity driven by high levels of vascular endothelial growth factor (VEGF) (Chaimani and Salanti, 2015). From a pharmacological perspective, VEGF does not merely promote neoangiogenesis; it actively suppresses immune surveillance by inhibiting dendritic cell maturation and promoting the accumulation of myeloid-derived suppressor cells (MDSCs) and regulatory T cells (Tregs) (Shigeta et al., 2020). Therefore, anti-angiogenic TKIs or monoclonal antibodies dual-function as immune modulators. By normalizing the tumor vasculature during the critical “window of tumor vascular normalization” and counteracting VEGF-mediated immunosuppression, these agents facilitate the infiltration of cytotoxic CD8^+^ T cells, thereby providing a primed microenvironment that maximizes the efficacy of PD-1/PD-L1 inhibitors (Kudo, 2020a). However, it is essential to recognize that these anti-angiogenic agents are not entirely equivalent; large-molecule anti-VEGF antibodies (e.g., bevacizumab) possess highly specific targets, whereas multi-target TKIs (e.g., sorafenib) have broader kinase inhibitory spectra and distinct toxicity profiles. This mechanistic rationale explains why combinations like atezolizumab plus bevacizumab and camrelizumab plus rivoceranib consistently outperform the single-target kinase inhibition of sorafenib in our network rankings. Furthermore, the high ranking of nivolumab plus ipilimumab (CheckMate 9DW) underscores the potency of dual checkpoint blockade (CTLA-4 and PD-1 inhibition) in overcoming T-cell exhaustion at distinct phases of the immune cycle, a strategy proving effective across multiple solid tumors. Selecting optimal first-line regimens is increasingly critical, as overcoming TME-mediated resistance and managing patients after initial ICI exposure remains a major challenge across various malignancies (Giudice et al., 2025; Song et al., 2025).

A novel aspect of our study is the integrated analysis of first-line and second-line trials. Traditionally, these settings are treated as distinct entities. Our updated subgroup analysis highlighted a substantial difference between treatment lines when sorafenib is used as the common reference (P = 0.0006). This finding reflects their distinct clinical contexts: first-line combinations show clear superiority over sorafenib in treatment-naïve patients, whereas second-line agents are evaluated in refractory populations. Consequently, comparing second-line agents directly to an active first-line standard yields a pooled HR closer to the null (Yau et al., 2020; Kudo, 2020b). From a pharmacological standpoint, the sustained efficacy of these agents in the refractory setting is largely attributable to the distinct kinase inhibitory profiles of subsequent therapies. For instance, acquired resistance to first-line sorafenib or lenvatinib is frequently mediated by the compensatory upregulation of alternative signaling pathways, such as the MET and AXL pathways. Cabozantinib acts as a potent inhibitor of these escape pathways in addition to VEGFR (Abou-Alfa et al., 2018). Similarly, regorafenib possesses a broader and more potent multi-kinase inhibition spectrum than its structural analog sorafenib, targeting angiogenic, stromal, and oncogenic receptor tyrosine kinases (e.g., TIE2, FGFR) (Bruix et al., 2017). By pharmacologically targeting these specific escape mechanisms, later-line TKIs effectively resensitize the tumor, which explains their robust performance in our second-line analysis (Bruix et al., 2017; Abou-Alfa et al., 2018).

Safety remains a critical consideration in treatment selection. Our analysis highlighted a distinct trade-off between efficacy and toxicity. The safety profiles of these regimens are directly linked to their respective mechanisms of action. TKI-based combinations (e.g., camrelizumab plus rivoceranib, lenvatinib plus pembrolizumab) ranked highest for efficacy but carry the highest risk of Grade ≥ 3 TRAEs because they aggregate the ‘on-target, off-tumor’ toxicities of potent VEGFR inhibition (such as hypertension, proteinuria, and hand-foot skin reaction) with the immune-related adverse events (irAEs) induced by breaking immune self-tolerance via PD-1 blockade. In contrast, pembrolizumab monotherapy (KEYNOTE-394) and the dual immunotherapy regimen (nivolumab plus ipilimumab) avoid the cumulative endothelial toxicity of TKIs, translating to relatively favorable safety profiles in terms of severe physical symptoms (Pinter et al., 2021b). This distinction is clinically relevant, especially for patients with borderline performance status or underlying liver dysfunction (Child-Pugh B), who may not tolerate the intense pharmacological burden of TKI combinations.

Heterogeneity in HCC trials is a well-recognized challenge. Our study noted a high prevalence of Hepatitis B Virus (HBV) infection in Asian-led trials (e.g., ORIENT-32, KEYNOTE-394) compared with global trials (e.g., SHARP, HIMALAYA). Recent meta-analyses have suggested that ICI-based therapies might be more effective in viral-HCC (HBV/HCV) than in non-viral HCC (e.g., NASH/NAFLD) (Hiraoka et al., 2022; Pfister et al., 2021). The “viral antigen presentation” hypothesis postulates that viral-induced tumors have higher immunogenicity and a distinct immune infiltration pattern, making them more responsive to PD-1 blockade (Ho et al., 2020). Although we did not perform a meta-regression on etiology due to data limitations, the high ranking of Asian-predominant trials in our ORR and PFS analysis supports the biological plausibility of this hypothesis.

Several limitations of this study must be acknowledged. First, the network relies on indirect comparisons between certain agents (e.g., regorafenib vs. atezolizumab plus bevacizumab), as head-to-head trials are lacking (Llovet et al., 2022). Second, the control arms varied between studies (placebo in second-line vs. sorafenib/lenvatinib in first-line), which we adjusted for in our network model by anchoring all treatments to sorafenib, but residual transitivity issues may persist (Palmer, 2020). Third, the definitions of “refractory” or “intolerant” varied slightly across second-line trials, which could introduce clinical heterogeneity (Salanti, 2012). Finally, we could not assess the impact of subsequent therapies, which are becoming increasingly common and may dilute the OS benefit in first-line trials (Reig et al., 2022).

In conclusion, our network meta-analysis confirms the therapeutic dominance of ICI-based combinations in advanced HCC. Using sorafenib as the standard of care reference, our study highlights the distinct relative efficacy profiles of active systemic therapies depending on their specific treatment setting (first-line versus second-line). While combination therapies offer the highest efficacy through pharmacological synergy, toxicity profiles and patient-specific factors such as etiology and liver function must guide personalized treatment sequencing (Casak et al., 2021; Haber et al., 2021). Future pharmacological research should focus on identifying predictive biomarkers to select patients who would benefit most from specific mechanistic combinations versus sequential monotherapies.

## Data Availability

The original contributions presented in the study are included in the article/Supplementary Material, further inquiries can be directed to the corresponding author.
