# Supplementary material for: Efficacy of immune-based combinations across treatment lines in advanced hepatocellular carcinoma: a systematic review and network meta-analysis
Source: Front Pharmacol. 2026 Jun 10;17:1837089. doi: 10.3389/fphar.2026.1837089 (PMC13290765; doi:10.3389/fphar.2026.1837089)
Supplement: Supplementary file 1 [file Supplementaryfile1.docx]

Supplementary Figures

| Search Step | Query |
| --- | --- |
| #1 Population | Carcinoma, Hepatocellular[Mesh] OR "Liver Neoplasms"[Mesh] OR "Hepatocellular carcinoma"[Title/Abstract] OR "Liver cancer"[Title/Abstract] OR HCC[Title/Abstract] |
| #2 Intervention | Sorafenib OR "Lenvatinib" OR "Regorafenib" OR "Cabozantinib" OR "Ramucirumab" OR "Pembrolizumab" OR "Atezolizumab" OR "Bevacizumab" OR "Nivolumab" OR "Ipilimumab" OR "Durvalumab" OR "Tremelimumab" OR "Camrelizumab" OR "Rivoceranib" OR "Sintilimab" OR "Apatinib" |
| #3 Study Design | Randomized Controlled Trial[Publication Type] OR "Randomized"[Title/Abstract] OR "Placebo"[Title/Abstract] |
| #4 Final Search | #1 AND #2 AND #3 |

Table S1: Detailed search strategy.

| Study | Treatment Arm | Total Patients (N) | Grade ≥3 TRAEs (n) | Rate (%) |
| --- | --- | --- | --- | --- |
| RESORCE | Regorafenib | 379 | 175 | 46.20% |
| Placebo | 194 | 24 | 12.40% |  |
| CELESTIAL | Cabozantinib | 467 | 316 | 67.70% |
| Placebo | 237 | 86 | 36.30% |  |
| REACH-2 | Ramucirumab | 197 | 68 | 34.50% |
| Placebo | 95 | 28 | 29.50% |  |
| KEYNOTE-394 | Pembrolizumab | 299 | 36 | 12.00% |
| Placebo | 153 | 9 | 5.90% |  |
| IMbrave150 | Atezolizumab + Bevacizumab | 329 | 143 | 43.50% |
| Sorafenib | 156 | 72 | 46.20% |  |
| REFLECT | Lenvatinib | 476 | 270 | 56.70% |
| Sorafenib | 475 | 231 | 48.60% |  |
| HIMALAYA | Durvalumab + Tremelimumab | 388 | 68 | 17.50% |
| Sorafenib | 374 | 37 | 9.90% |  |
| ORIENT-32 | Sintilimab + IBI305 | 380 | 110 | 28.90% |
| Sorafenib | 185 | 30 | 16.20% |  |
| CARES-310 | Camrelizumab + Rivoceranib | 272 | 220 | 80.90% |
| Sorafenib | 269 | 141 | 52.40% |  |
| CheckMate 9DW | Nivolumab + Ipilimumab | 332 | 137 | 41.30% |
| Lenvatinib/Sorafenib | 325 | 138 | 42.50% |  |
| LEAP-002 | Lenvatinib + Pembrolizumab | 395 | 243 | 61.50% |
| Lenvatinib + Placebo | 395 | 224 | 56.70% |  |
| SHARP | Sorafenib | 297 | 24 | 8.10% |
| Placebo | 302 | 4 | 1.30% |  |

Table S2: Detailed Safety Data .


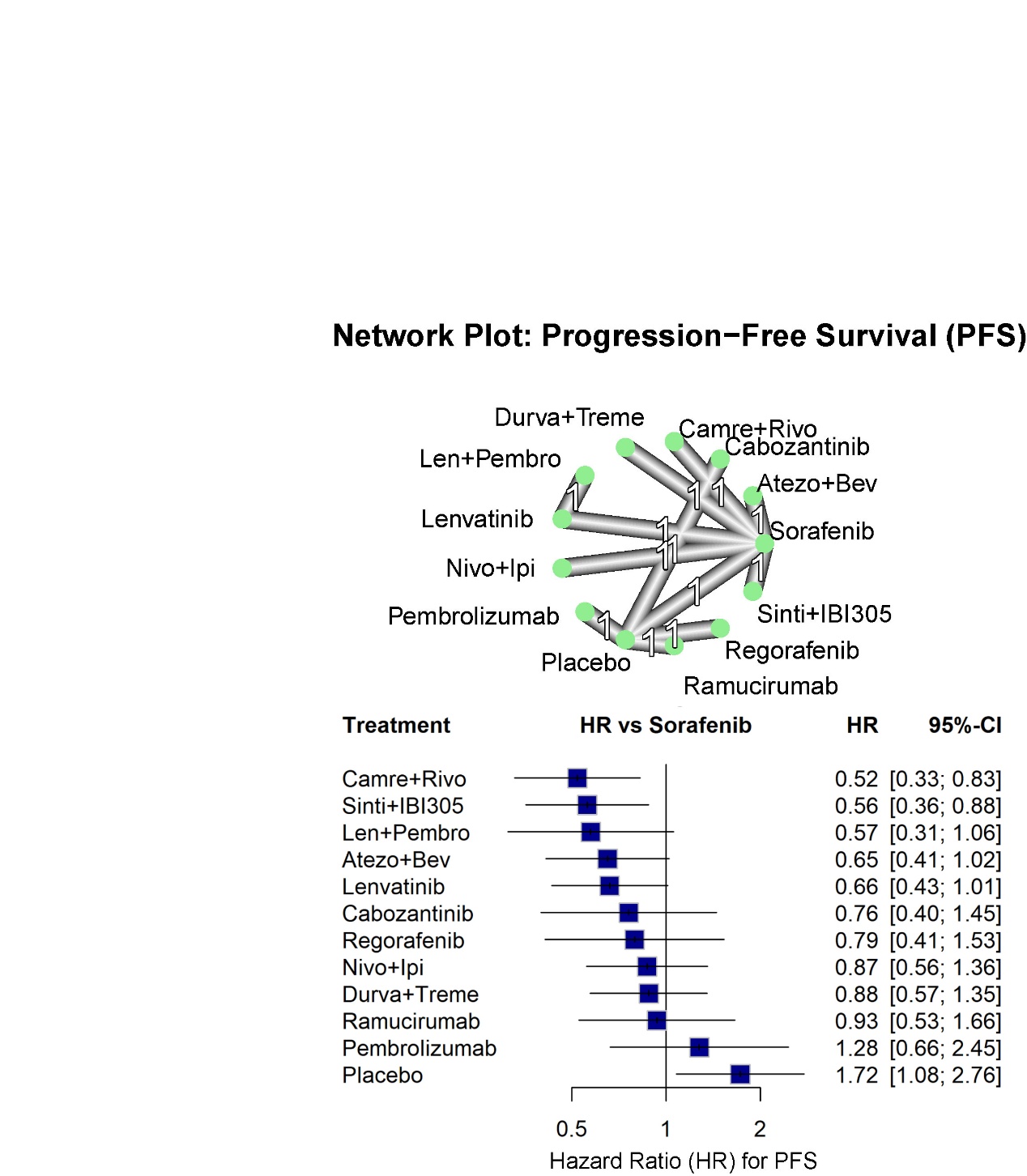


Figure S1. Network and Forest Plot for Progression-Free Survival (PFS)

(A) Network geometry for PFS. (B) Forest plot of HRs for PFS compared with Sorafenib. HR < 1 indicates a reduction in the risk of disease progression or death.


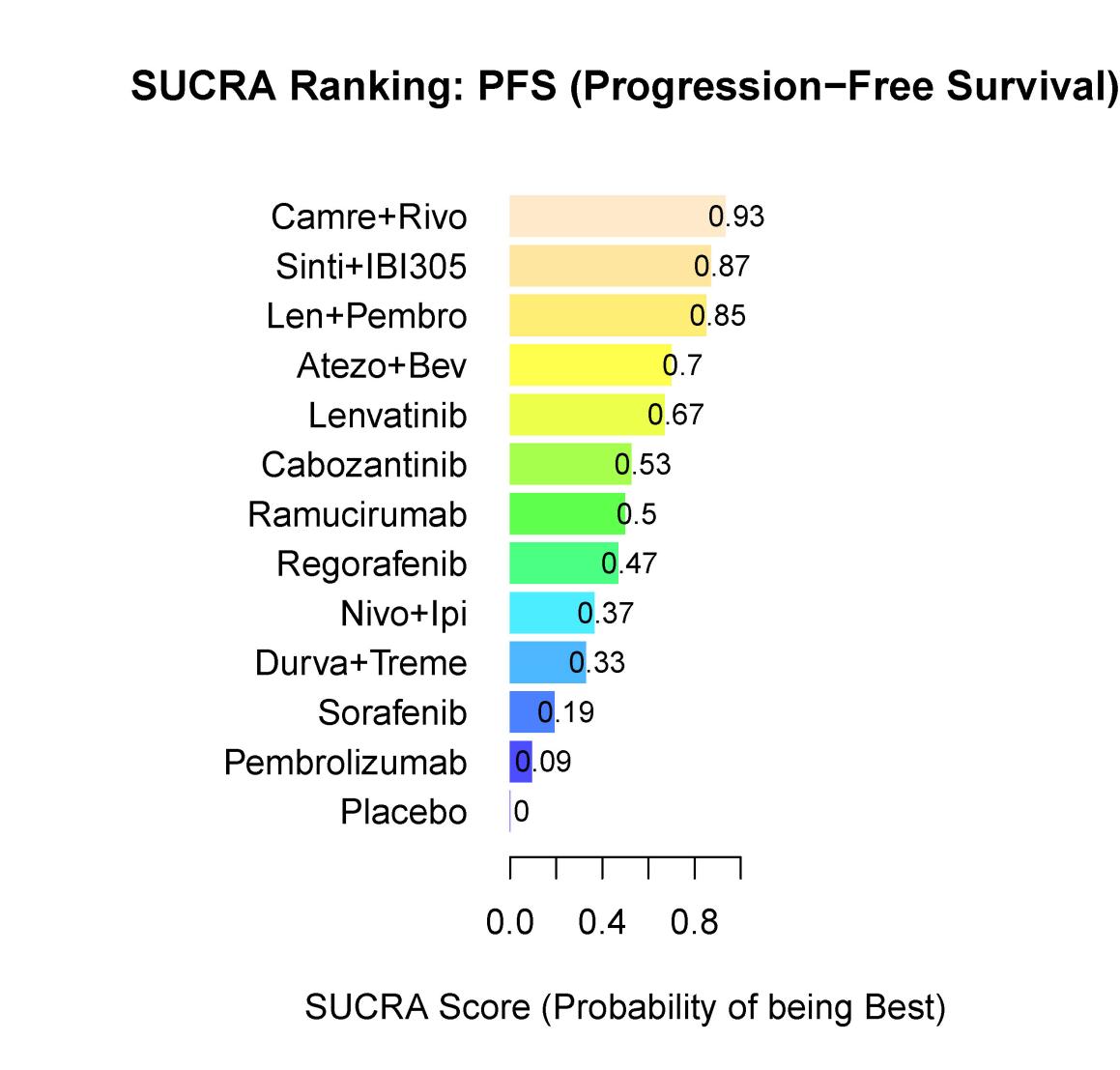


Figure S2. SUCRA Ranking for Progression-Free Survival

Ranking of treatments for PFS based on SUCRA scores. Higher scores indicate better efficacy in delaying tumor progression.


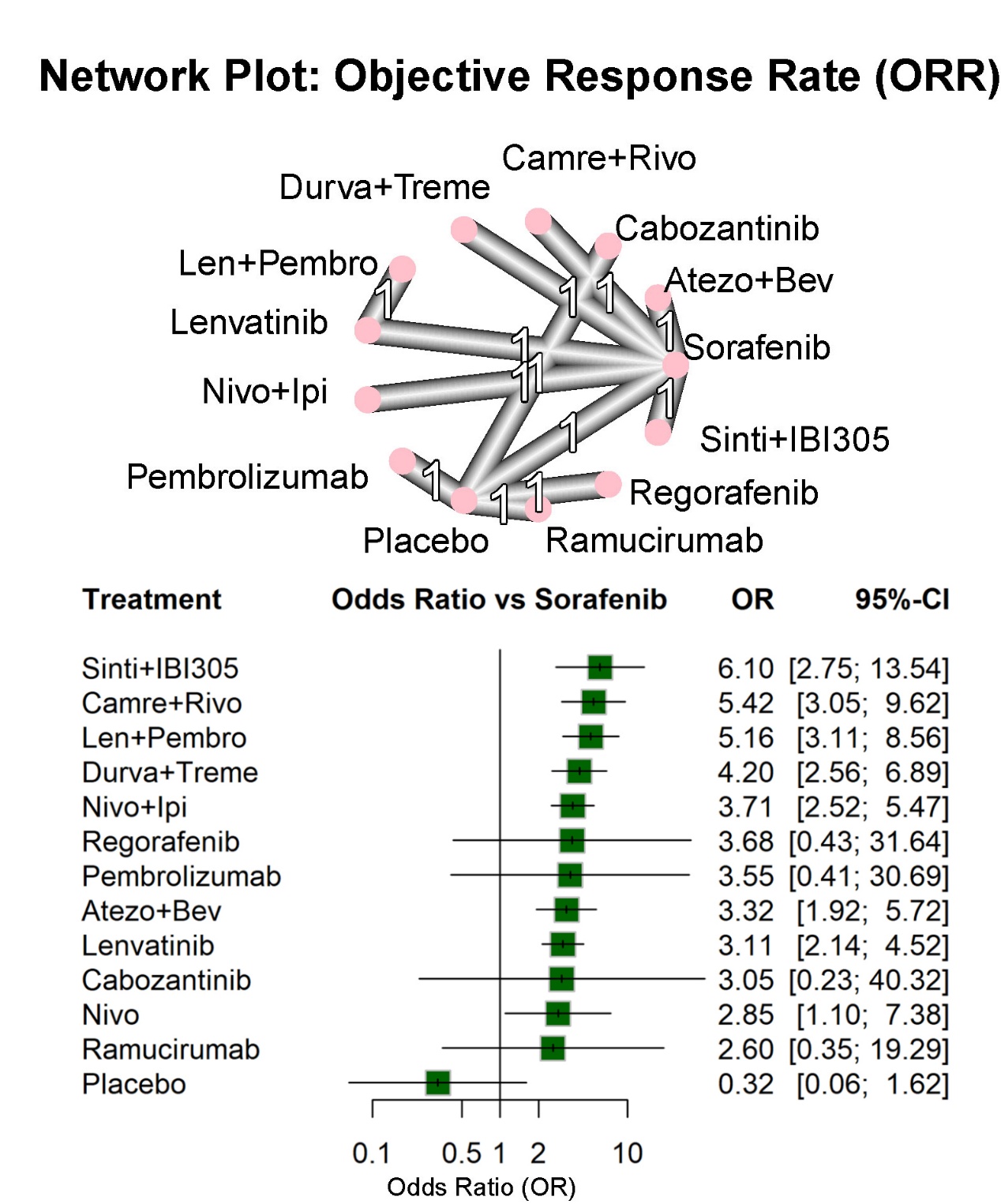


Figure S3. Network and Forest Plot for Objective Response Rate (ORR)

(A) Network geometry for ORR. (B) Forest plot of Odds Ratios (ORs) for ORR compared with Sorafenib. OR > 1 indicates a higher likelihood of achieving a tumor response.


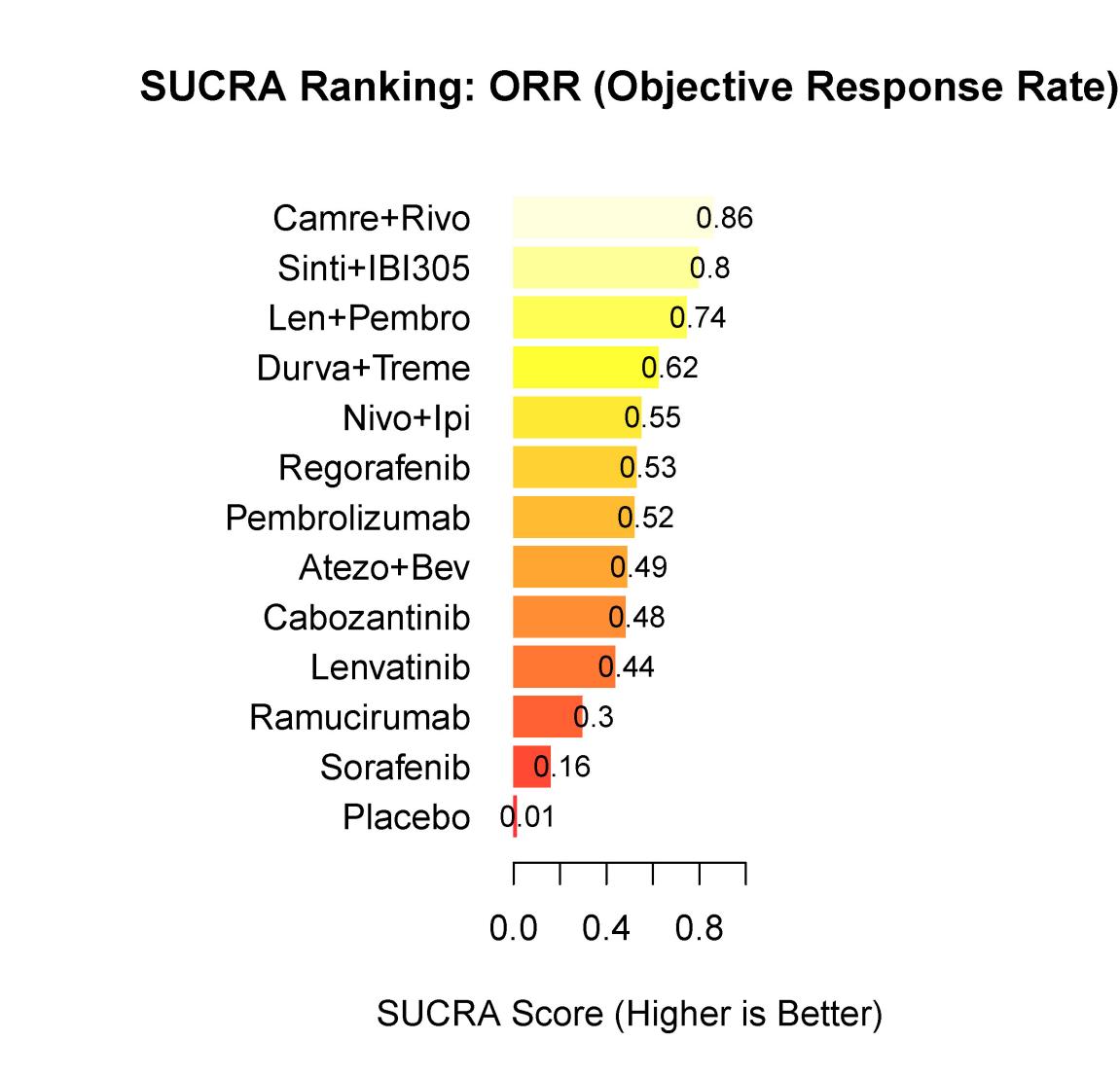


Figure S4. SUCRA Ranking for Objective Response Rate

Ranking of treatments for ORR. Higher SUCRA scores indicate a greater probability of achieving tumor shrinkage.


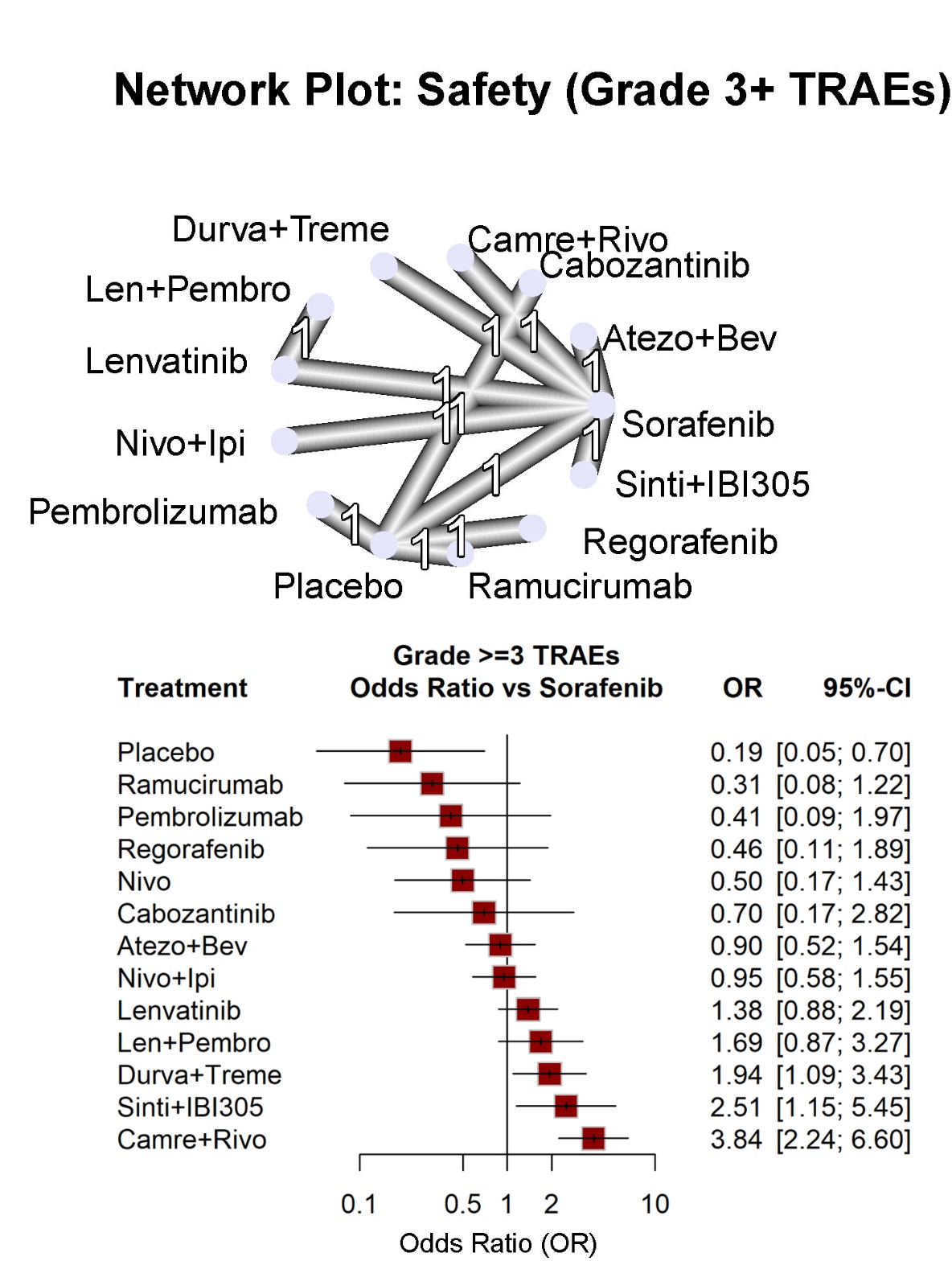


Figure S5. Network and Forest Plot for Safety (Grade ≥3 TRAEs)

(A) Network geometry for safety analysis. (B) Forest plot of Odds Ratios (ORs) for Grade ≥3 Treatment-Related Adverse Events (TRAEs) compared with Placebo. OR < 1 indicates a lower risk of severe adverse events.


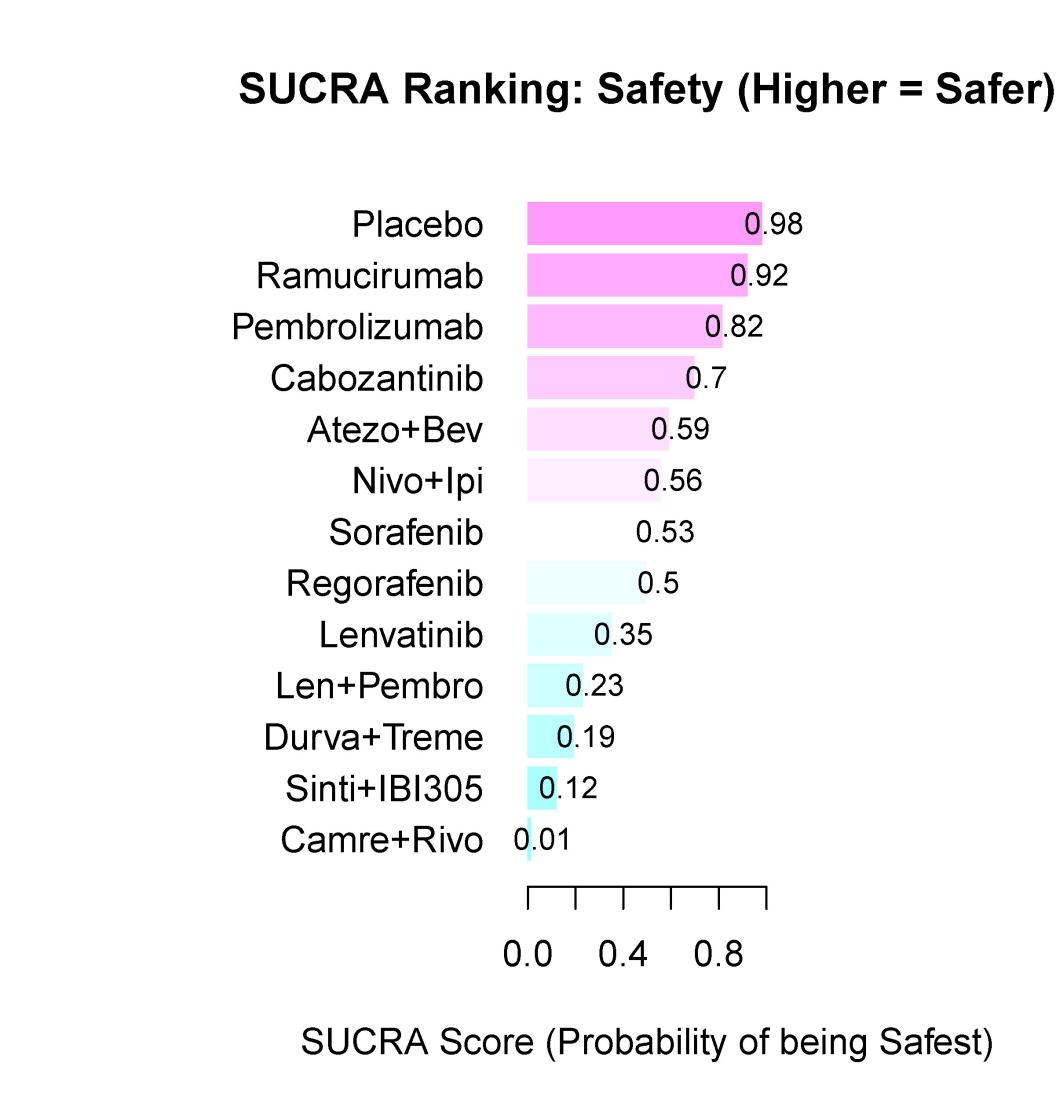


Figure S6. SUCRA Ranking for Safety

Ranking of treatments based on safety profile. Note: For safety outcomes, higher SUCRA scores indicate a better safety profile (i.e., lower risk of adverse events). Placebo ranks highest, as expected.


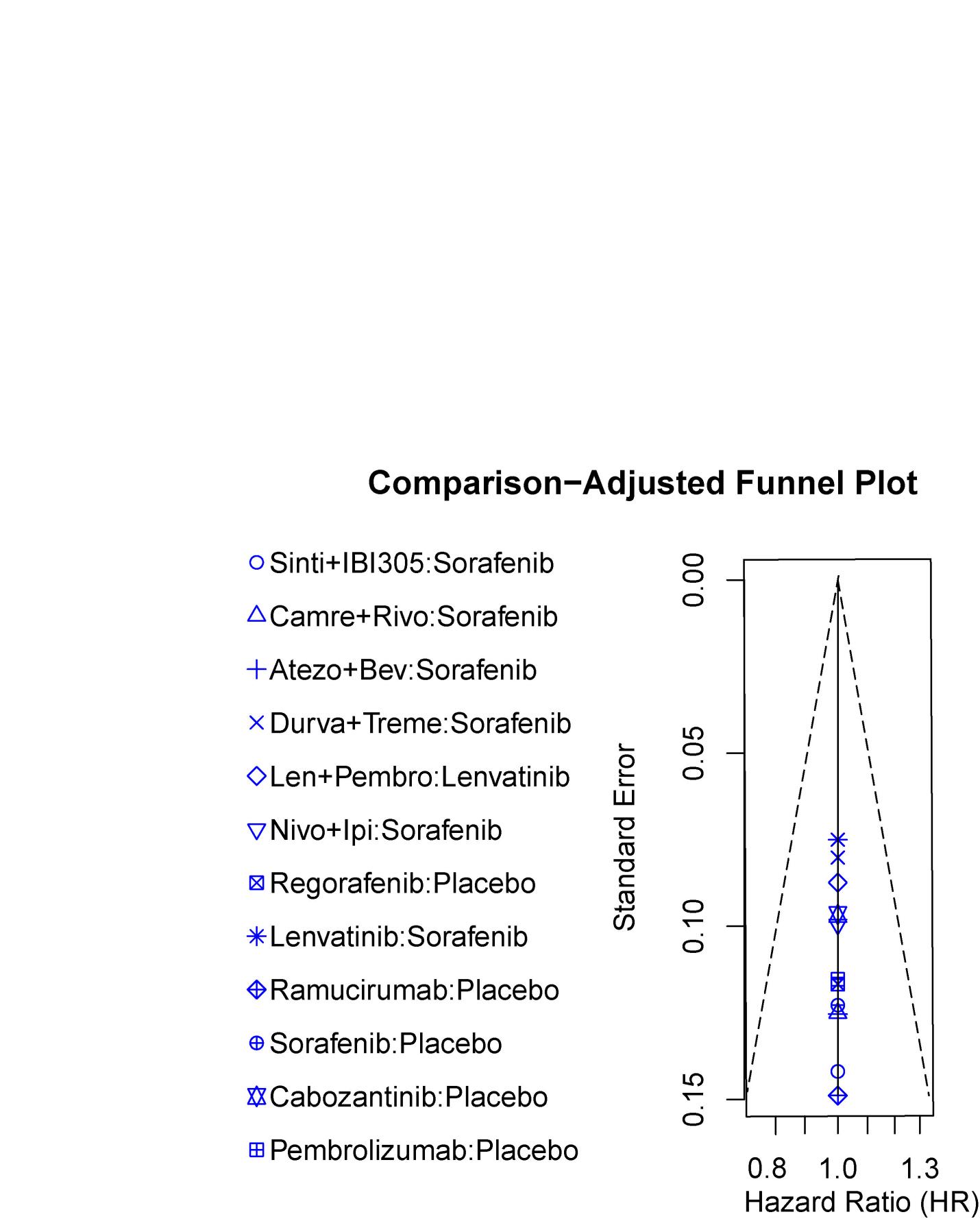


Figure S7. Comparison-Adjusted Funnel Plot

Comparison-adjusted funnel plot for overall survival to assess publication bias. The vertical line represents the null effect, and the dots represent individual study comparisons. Symmetry around the axis generally suggests a low risk of small-study effects or publication bias. The centering of points indicates consistency within the star-shaped network structure.
